# New Invasive Nemertean Species (*Cephalothrix Simula*) in England with High Levels of Tetrodotoxin and a Microbiome Linked to Toxin Metabolism

**DOI:** 10.3390/md16110452

**Published:** 2018-11-16

**Authors:** Andrew D. Turner, David Fenwick, Andy Powell, Monika Dhanji-Rapkova, Charlotte Ford, Robert G. Hatfield, Andres Santos, Jaime Martinez-Urtaza, Tim P. Bean, Craig Baker-Austin, Paul Stebbing

**Affiliations:** 1 Centre for Environment, Fisheries and Aquaculture Science (Cefas), Barrack Road, Weymouth, Dorset DT4 8UB, UK; Andy.powell@cefas.co.uk (A.P.); monika.dhanjirapkova@cefas.co.uk (M.D.-R.); Charlotte.ford@cefas.co.uk (C.F.); Robert.hatfield@cefas.co.uk (R.G.H.); andres.santos@ucl.cl (A.S.); jaime.martinez-urtaza@cefas.co.uk (J.M.-U.); Tim.Bean@cefas.co.uk (T.P.B.); craig.baker-austin@cefas.co.uk (C.B.-A.); Paul.stebbing@cefas.co.uk (P.S.); 2 Roscadghill Parc, Penzance, Cornwall TR18 3QY, UK; davidfenwicksnr@googlemail.com; 3 Laboratory of Applied and Molecular Biology, Avenida Alemania 0458, 4810296 Temuco, Chile; 4 Scientific and Technological Bioresource Nucleus (BIOREN), Universidad de La Frontera, Avenida Francisco Salazar 01145, 4811230 Temuco, Chile

**Keywords:** tetrodotoxin, nemertean, bacteria, toxicity, invasive species

## Abstract

The marine nemertean *Cephalothrix simula* originates from the Pacific Ocean but in recent years has been discovered in northern Europe. The species has been associated with high levels of the marine neurotoxin Tetrodotoxin, traditionally associated with Pufferfish Poisoning. This study reports the first discovery of two organisms of *C. simula* in the UK, showing the geographical extent of this species is wider than originally described. Species identification was initially conducted morphologically, with confirmation by Cox 1 DNA sequencing. 16S gene sequencing enabled the taxonomic assignment of the microbiome, showing the prevalence of a large number of bacterial genera previously associated with TTX production including Alteromonas, *Vibrio* and Pseudomonas. LC-MS/MS analysis of the nemertean tissue revealed the presence of multiple analogues of TTX, dominated by the parent TTX, with a total toxin concentration quantified at 54 µg TTX per g of tissue. *Pseudomonas luteola* isolated from *C. simula*, together with *Vibrio alginolyticus* from the native nemertean *Tubulanus annulatus*, were cultured at low temperature and both found to contain TTX. Overall, this paper confirms the high toxicity of a newly discovered invasive nemertean species with links to toxin-producing marine bacteria and the potential risk to human safety. Further work is required to assess the geographical extent and toxicity range of *C. simula* along the UK coast in order to properly gauge the potential impacts on the environment and human safety.

## 1. Introduction

Nemerteans—or ribbon worms—are a diverse family of benthic unsegmented invertebrates, the majority of which are found in the marine environment. Up to 1200 species are known, existing throughout the world, in a wide variety of habitats, although in the marine environment usually located between intertidal and deep-sea environments. They have been found amongst rocks, or buried in mud or sand, or alternatively found amongst algae or plant material. They are known to feed on smaller invertebrates, including bivalve molluscs [1,2] but with some species feeding on plant and planktonic species [3,4,5,6]. The nemerteans possess a unique proboscis structure, which is primarily used for capture of prey. A range of chemicals are known to be produced by the proboscis as part of the prey capture process as well as a predator deterrent [4,5,7,8,9,10,11,12,13,14]. Some species of nemertean have been shown to exhibit high levels of neurotoxicity, with [15] first detecting Tetrodotoxin (TTX) in the nemertean species *Lineus fuscoviridis* and *Tubulanus punctatus*.

TTX, along with a range of analogues together termed the tetrodotoxins (TTXs) is a low molecular weight, water soluble neurotoxin associated with neurotoxic marine poisonings [16] found in a wide range of marine phyla, including most famously the pufferfish, as well as gastropods, crustacean, octopus and echinoderms [15,17,18,19,20,21,22,23,24,25,26,27,28,29,30,31,32,33,34,35]. In recent years it has been detected in bivalve molluscs such as mussels, oysters and clams from the UK [36,37] as well as other parts of Europe including Greece, the Netherlands and Spain [38,39] and also New Zealand [40]. Unlike other marine biotoxins, TTX is associated primarily in the literature with production by bacterial species [19,41,42,43,44], although the biosynthetic pathway for the toxin has not yet been elucidated [42] and reports proposing association with phytoplankton have also been published for example, [30,38]. Most notable is the co-existence of TTX with certain genera of bacteria, including *Vibrio*, *Bacillus* and *Pseudomonas* [35,36,42,43,44]. The relationship between TTX and Vibrio is especially important given the links between bacterial prevalence, toxin occurrence and climatic change [37,45,46,47,48,49,50,51,52].

The occurrence of TTX in the ribbon worms of genus *Cephalothrix* (species *Cephalothrix linearis*) was first reported from intertidal zones in Japan during 1987 and 1988 [53]. Neurotoxicity was later reported from the same species in Hiroshima Bay, Japan by mouse bioassay (MBA), subsequently characterising TTX and the associated analogues 4-epi TTX and anhydro TTX using High Performance Liquid Chromatography (HPLC) methods [54]. These highly toxic nemerteans were found attached to aquacultured oysters, thus presenting a potential food safety risk. Following surveillance activities, around 80% of the specimens of *Cephalothrix simula* collected in Japan were found to be toxic [14]. Furthermore, authors have demonstrated that other nemerteans are not generally found to be toxic, with only *Cephalothrix* shown to possess very high levels of neurotoxicity [14]. More recently, there have been reports of the presence of the TTX-producing bacteria *Bacillus* sp. 1839 in *C. simula* isolated from the Sea of Japan [55], fitting with the findings of other authors who have established that these species and other species of bacteria such as *Vibrio alginolyticus* may play an important role in either the production or accumulation of toxin within nemerteans [23,56,57,58,59,60,61]. 

Whilst the origin of *C. simula* appears to have been the north west Pacific Ocean, it has in more recent years been discovered within European waters [62]. The species has been found along the Atlantic and Mediterranean coasts of Spain and along the Adriatic coast of Italy [63,64,65]. Multiple findings were also reported throughout Spain and Portugal between 2013 and 2015 [66]. The first and to date, only record of *C. simila* in northern Europe was published by [67], who identified the species using DNA sequencing during biodiversity surveys in the Netherlands. The potential presence of TTX and *Vibrio* bacteria was also investigated in seven species of British nemerteans, including the only native *Cephalothrix* species, *C. rufifrons* [68]. Tentative identification of *Vibrio alginolyticus* was reported, with HPLC showing chromatographic peaks indicative of TTXs. However, to date, there has been no conclusive proof of TTX-presence in British ribbon worms.

This paper reports on the first recorded detection of *Cephalothrix simula* in the UK and subsequent investigations conducted in relation to molecular confirmation of the species, toxin testing using validated liquid chromatography with tandem mass spectrometry (LC-MS/MS) methods and the assessment and toxin content of bacterial strains. The potential impacts that these findings may have are discussed in addition to speculation on how the species may have been introduced.

## 2. Results

### 2.1. Samples

**Figure 1:**
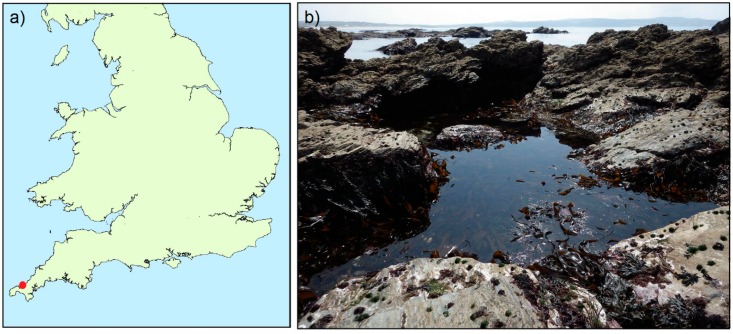
Maps showing (**a**) geographical location within the UK (**b**) photograph of low tide pool containing nemerteans from this study.

In May 2018, a headland west of Godrevy Point, near Hayle, SW Cornwall (UK grid reference SW 57935 43200), was visited at low water during an extra low spring tide (Figure 1). During a general biodiversity survey, searches were focused on reasonably small areas, including overhangs and zones just above and just below the low water mark. In one pool—exposed on the extreme lowershore—previously found to contain the Pacific non-native species *Perophora japonica* and *Pikea californica*, two nemerteans tentatively resembling the native nemertean *Emplectonema gracile* were discovered. The two organisms were found in a single knot under an empty mussel valve, fixed underneath a rock within the darker northern side of a large pool. The two specimens were separated and immediately photographed using a Canon 5D MkII, Canon MP-E 65 mm lens and Canon MT-24EX twin flash on various acrylic and naturalistic backgrounds in filtered seawater. Each specimen was then placed in a plastic container containing filtered seawater and refrigerated at around 10 °C. The specimens were kept alive before shipping next day delivery to the Cefas laboratory in Weymouth. The specimens were shipped in plastic tubes one containing foam soaked in oxygenated filtered seawater and the other containing 95% ethanol (Table 1). Seawater was filtered using 12.5 cm dia. plastic buchner funnel and qualitative filter paper to remove organisms that might have contaminated the sample given at least part of one nemertean would be required for sequencing. Eleven days later, two native nemertean species (*C. rubifrons* and *Tubulanus annulatus*) were collected and shipped to Cefas as described above (Table 1).

**Figure 2:**
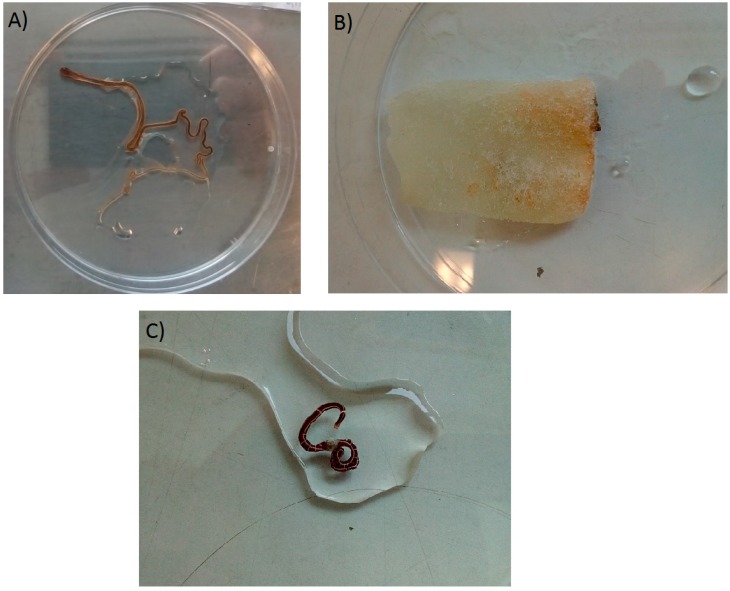
Samples received for testing, (**A**) tentative *C. simula*; (**B**) *C. rubifrons* (on sponge); (**C**) *T. annulatus.*

On receiving the specimens (Table 1) all animals were photographed and measured (Figure 2). For the two suspected *C. simula*, two organisms were received (Table 1, worm 1a and 1b). Worm 1a sent in seawater was long, approximately 30 cm uncoiled, whilst worm 1b, preserved in ethanol, was much shorter, approximately 20 mm in total length. Worms 2 and 3 were each approximately 10 cm in length. The nemerteans for bacterial and toxin testing (worms 1a and 3) were each homogenised separately in a sterile petri-dish using a sterile razor blade. Sub-samples of the homogenised tissue were taken for microbiological testing, with the rest subjected to toxin analysis. The sample containing *C. rubifrons* (Table 1, worm 2; Figure 2) found to contain dead organisms on the sponge insert with evidence of some dark coloured decay, were treated differently. The sponge was halved using a sterile razor blade, with one half taken from microbial testing and the other half utilised for toxin analysis.

### 2.2. Species Identification

The Cox 1 DNA barcode sequence of worm 1b (Table 1) confirmed the tentative morphological identity of *Cephalothrix simula*. Both fragments of worm amplified successfully and provided 290 bp of useable, identical, Cox 1 sequence, which had 100% identity to the previously sequenced *C. simula* voucher specimen ([67]; Genbank accession number KP411247.1). In addition, the sequenced samples had only 86% sequence identity to the morphologically similar species *C. rufifrons*. A blank (control) DNA extract did not amplify via PCR and a positive metazoan (control) extract was correctly identified as a different metazoan species. The sequences are available as Supplementary Figure S1.

### 2.3. Microbiology

*C. simula* yielded a pure culture from an ASPW enrichment broth incubated at 44 °C for 24 h, sub-cultured onto TCBS that was subsequently incubated at 22 °C for 48 h. 5 colonies were sub-cultured on to Marine agar and identified using biochemical analysis API20E (Biomerieux, Marcy l’Etoile, France). These isolates were identified as bacilli, *Pseudomonas lutiola*. *Vibrio algionolyticus* was isolated from *C. rufifrons,* recovered from an enrichment broth of ASPW at 44 °C for 24 h. This was sub-cultured onto TCBS incubated at 37 °C for 24 h. Isolates were transferred to Marine agar and identified using biochemical analyses API20E (Biomerieux, Marcy l’Etoile, France) and PCR using species-specific PCR assay.

### 2.4. Microbiome

A metagenomic approach was used to characterize the total microbial community present in the samples. A total of 82,342 reads was generated by the MinION sequencer, with a quality average of 9.55 and a sequence length average of 1350 pb. The vast majority of the reads taxonomically assigned based on the 16S gene sequence (86.05%) belonged to the Bacteria domain and only 0.1% of reads were identified as Archaea. These reads were classified into 72 classes, 152 orders, 324 families, 866 genera and 2054 species. The remaining reads (13.95%) were not assigned to any known taxa.

**Figure 3:**
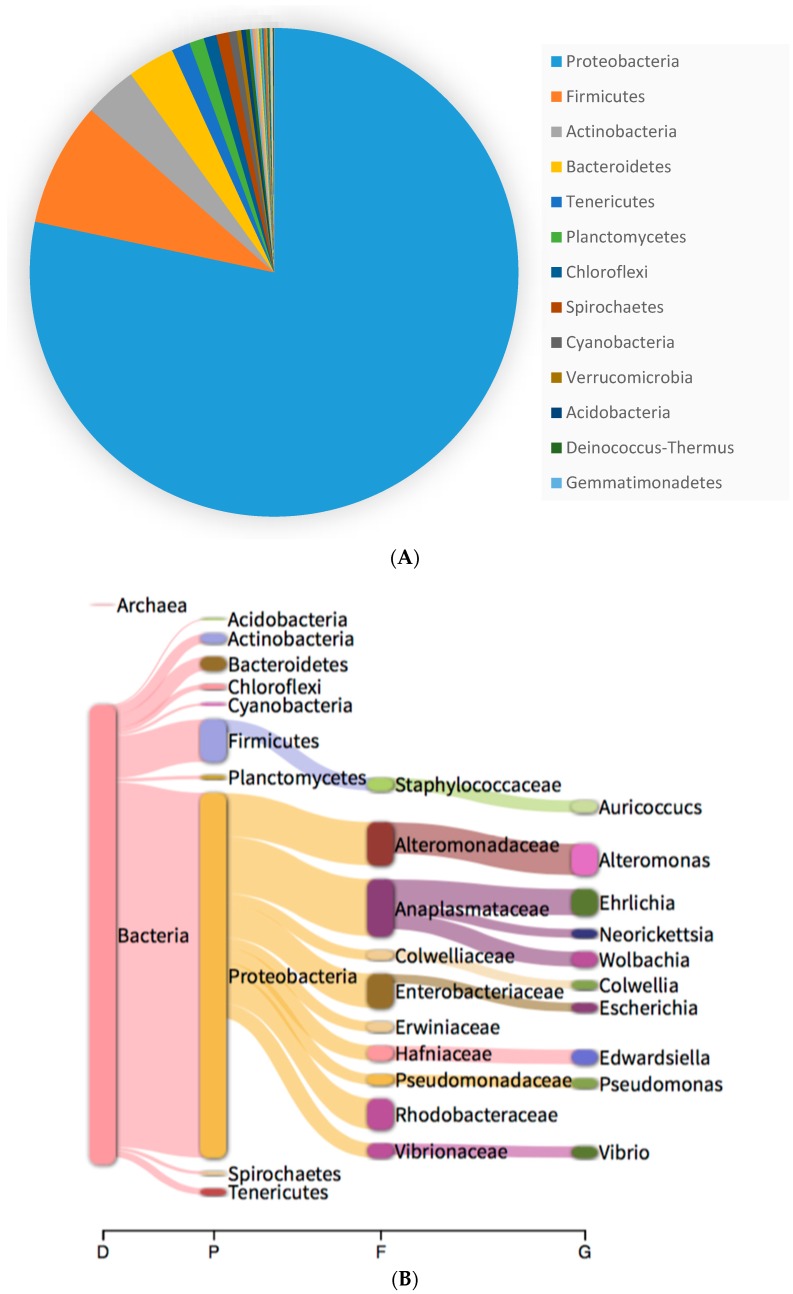
Bacterial taxonomic profile determined at (**A**) phylum and (**B**) genus level for *C. simula* sample following sequencing using MinION platform.

Analysis of the *C. simula* microbiome identified Proteobacteria as the most prevalent phylum with a total number of reads of 48,211 (81.15%), followed by Firmicutes with 5247 (8.82%) Actinobacteria with 1498 (2.52%) and Bacteroidetes with 1116 (2.32%) (Figure 3a). The most abundant orders among bacteria were Rickettsiales (10,259 reads: 17.27%) Enterobacterales (8833 reads: 14.87%), Alteromonadales (7780 reads: 13.09%), Rhodobacterales (4754 reads: 8%), Pseudomonadales (2899 reads: 4.88%) Bacillales (2586 reads: 4.35%) and Vibrionales (2141 reads: 3.6%) (Figure 3b).

At genus level, the most abundant were *Alteromonas* (2066: 5.8%), *Ehrlichia* (1666: 4.1%), *Edwadsellia* (985: 2.94%), *Vibrio* (788: 2.55%) and *Wolbachia* (954: 2.52%) (Table 2). Only a small number of these genera could be classified at the species level, identifying *Ehrlichia canis* (1455: 3.29%), *Edwarsiella ictaluri* (979: 2.7%), *Escherichia coli* (618: 1.21%)*, Aurococcus indicus* (770: 1.82%) and *Mediterranean alteromonas* (740: 1.78%) as the most abundant species.

Within the *Alteromonas* genus, the species with the highest relative abundance were *Alteromonas mediterranea* (1.7%), *Alteromonas naphthalenivorans* (1.4%) and *Alteromonas macleodii* (0.7%), while for pseudomonas genus were *Pseudomonas aeruginosa* (0.8%), *Pseudomonas citronellolis* (0.37%) and *Pseudomonas putida* (0.27%). Regarding Vibrio, the most abundant species were *V. corallilyticus* (0.80%), *V. harveyi* (0.72%), *V. cholerae* (0.51%) *V. parahaemolyticus* (0.22%) and *V. owensii* (0.22%).

A relatively small number of reads were assigned to Enterobacteria, including several species associated with faecal contamination and human pathogens. The species identified included *Escherichia coli* (618—1.9%), *Salmonella enterica* (327—0.7%), *Klebsiella pneumoniae* (113—0.3%).

### 2.5. Toxin Analysis of Tissue

**Figure 4:**
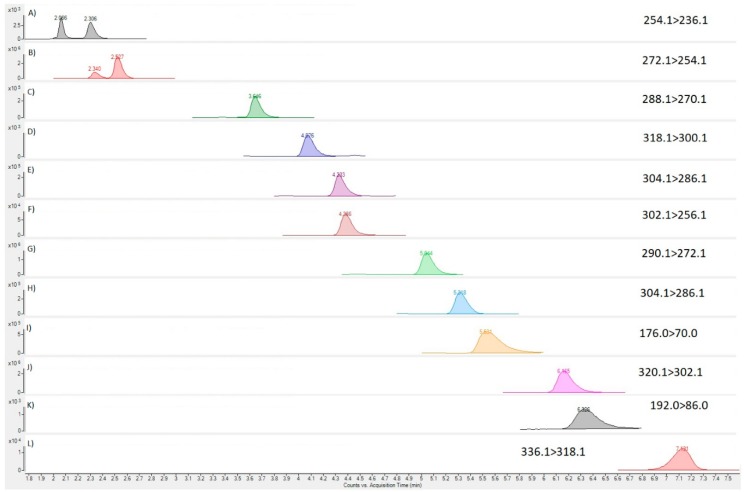
MRM chromatograms for TTX analogues in QC sample; (**A**) 4,9-anhydro-5,6,11-trideoxy TTX; (**B**) 5,6,11-trideoxy TTX; (**C**) 6,11-dideoxy TTX; (**D**) 4,9-anhydro-11-oxo TTX; (**E**) 5-deoxy TTX; (**F**) 4,9-anhydro TTX; (**G**) 11-nor TTX-6-ol; (**H**) 11-deoxy TTX; (**I**) arginine; (**J**) TTX; (**K**) OH-arginine; (**L**) 11-oxo TTX.

**Figure 5:**
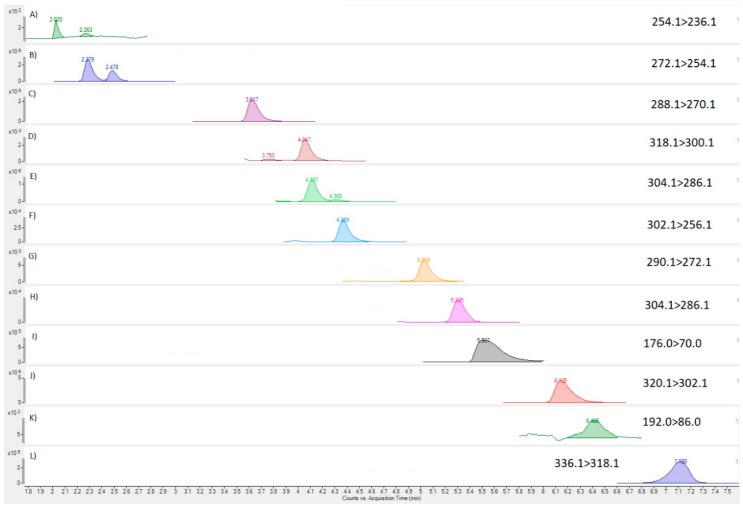
MRM chromatograms for TTX analogues in nemertean sample; (**A**) 4,9-anhydro-5,6,11-trideoxy TTX; (**B**) 5,6,11-trideoxy TTX; (**C**) 6,11-dideoxy TTX; (**D**) 4,9-anhydro-11-oxo TTX; (**E**) 5-deoxy TTX; (**F**) 4,9-anhydro TTX; (**G**) 11-nor TTX-6-ol; (**H**) 11-deoxy TTX; (**I**) arginine; (**J**) TTX; (**K**) OH-arginine; (**L**) 11-oxo TTX.

LC-MS/MS analysis of the invasive *C. simula* nemertean extract (Worm 1a) was conducted to assess the presence of TTX and its associated analogues in comparison with those identified in our internal quality control (QC) sample (Figure 4). Multiple reaction monitoring (MRM) transitions were detected for many of the analytes incorporated into the detection method, with the positive identification of TTX, 5,6,11-trideoxy TTX, 11-nor TTX-6-ol, 4,9-anhydro TTX, 5-deoxy TTX, 11-deoxy TTX, 4,9-anhydro-5,6,11-trideoxy TTX and 11-oxo TTX (Figure 5). Chromatographic results evidenced the effective resolution between TTX and the matrix co-extractives, arginine and hydroxy-arginine. When quantified against the external TTX calibration, the sum of all TTX analogue concentrations was found to be 54.3 µg per gram of tissue. The parent TTX was the dominant analogue, corresponding to 64% of the total toxin concentrations, followed by 6,11-dideoxy TTX (21%), 5,6,11-trideoxy TTX (9%) and 11-oxo TTX (5%), with all other analogues present at <1% of the total toxin abundance (Table 3). LC-MS/MS analysis was also conducted for Paralytic Shellfish Toxins (PST) following the method of [69] but no PST analogues were detected. Analysis of Worm 2 and Worm 3 samples revealed the absence of any chromatographic peaks indicative of either TTXs or PSTs (data not shown).

### 2.6. Toxin Analysis of Bacterial Cultures

**Figure 6:**
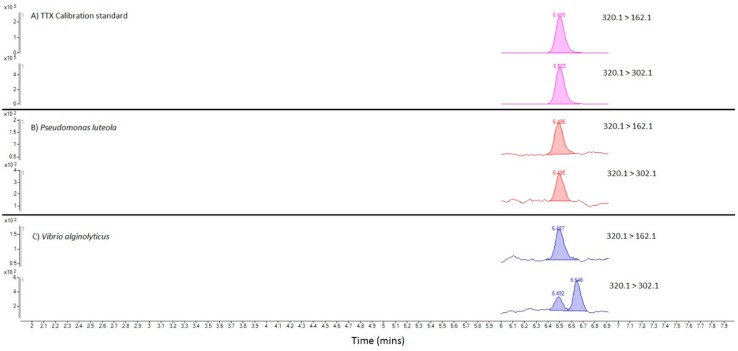
MRM chromatograms for TTX in (**A**) TTX calibration standard; (**B**) SPE-cleaned extract of *P. luteola* isolated from *C. simula*; (**C**) SPE-cleaned extract of *V. alginolyticus* isolated from *C. rufifrons*.

Following the initial culturing of bacteria isolated from the toxic *C. simula* and grown at both 37 °C and 41.5 °C, LC-MS/MS showed the absence of any TTXs in any of the bacterial extracts. LC-MS/MS analysis was subsequently conducted of the cleaned-up bacterial pellet extracts isolated following low temperature (22 °C) culturing of *P. luteola* from the highly toxic *C. simula* sample (worm 1a) and *V. alginolyticus* from the non-toxic native nemertean species (worms 2 and 3). MRM peaks corresponding to both TTX transitions were identified with the same retention time as those for TTX in the calibration standard solutions in both sample 1a and sample 2 (Figure 6). The ion ratios between the two MRM transitions were within 5% of the ratio determined in the analytical standard, providing further confirmation of toxin presence. No TTX was detected in sample 3, corresponding to *V. alginolyticus* isolated from *T. annulatus.* TTX was the only tetrodotoxin analogue identified in any of the bacterial culture isolates. Quantitation conducted for both TTX-positive samples showed 93 ng and 88 ng TTX per litre of culture in sample 1a and sample 2 respectively.

Further investigations were conducted targeting mass culturing of bacteria for toxin production. Pure isolations were sub-cultured onto 4 Marine agar plates per isolate and incubated at 30 °C for 24 h. The bacterial growth from these plates were inoculated into 225 mL ASPW and 100mL NB and incubated for 24 h at 22 °C, 37 °C and 41.5 °C. Unfortunately, none of the incubations were found to contain toxins.

## 3. Discussion

### 3.1. Invasive Species Implications

With the spread of the invasive ribbon worm *C. simula* from the Pacific [62] into southern Europe [63,64,65,66] and more recently the emergence in northern Europe [67], it was considered likely that the species would become introduced into the UK in time. Although this is the first published record of the species being found within UK waters, historically there has been limited effort to monitor for the *C. simula* specifically, especially on a national scale. Routine ecological monitoring programmes within the UK have previously reported the presence of *Cephalothrix* spp. but identification of specimens is to genus level only. The identification of nemertean species is complex, relying historically on histological examination of internal anatomy and external morphology [70], usually based on a series of standardised characteristics [71]. These approaches have more recently been supplemented by molecular detection methods [70,72,73,74]. Such methods are important in order to distinguish between non-native species such as *C. simula and Cephalothrix* already recorded in the UK, *Cephalothrix arenaria* Hylbom, 1957, *Cephalothrix filiformis* Johnston, 1828, *Cephalothrix linearis* Rathke, 1799 and *Cephalothrix rufifrons* Johnston, 1837. Neither the traditional histological examination or the more recent molecular tools are applied as standard to statutory bio-diversity monitoring of the marine environment. This significantly limits the possible early detection of introduced nemertean species [67]. These limitations may have led to the species being previously missed or not adequately identified and therefore the current distribution of *C. simula* within the UK may be more extensive than currently recognised. Although to date only 2 specimens have been found, a population may be established in at least the local vicinity of the point of detection. Determining establishment and the distribution of the species in country will require dedicated monitoring, preferably applying molecular tools, given complexities in identification as discussed.

In recent years numerous species new to the UK have arrived along the south coast, including non-native species and southern species increasing their northern range. These include the tunicate Creeping Sea Squirt, *Perophora japonica*, the Pacific red alga *Pikea californica*, the opisthobranchs *Hermaea cantabra* and *Hermaea paucicirra,* the red algal parasite of *Gelidium*, *Gelidiocolax margaritoides the green alga Anadyomene stellata and* a number of members of the phylum Nemertea, such as *Lineus grubei*, *Nemertopsis bivittate* and *Vieitezia luzmurubeae* [75,76,77]. This highlights the south coast of England as a hotspot for introductions, with a combination of pathway intensity, natural dispersal [78] and climate change [79] contributing to the influx of species. In the case of *C. simula*, ref. [62] speculated that introduction may have occurred via ballast water, ship fouling, or aquaculture. The potential risk of introduction posed from ship fouling and ballast water in the UK has been highlighted [78], whilst aquaculture is considered less of a risk due to movement restrictions imposed on live animals into the country. Hydrological links between the Bay of Biscay and southern England. Ref. [80] further highlight the potential for this species to disperse via current movements and natural dispersal from other established populations. It is, however, difficult to speculate as to how or where *C. simula* was first introduced into the UK without a more detailed understanding of its current distribution. A propensity for rapid dispersal of the species has been suggested, so it is unlikely that a point of initial introduction will be identified [67].

There are no studies conducted on the potential impact that *C. simula* may have on its introduced range. Ref. [67] speculate that as a predator the species may affect local food chains. *C. simula* has been found to account for 28% of all nemerteans sampled at one of two Mediterranean sites [64], with no native *Cephalothrix* spp. reported, which could be as a result of competitive exclusion but there is a lack of quantitative data to support this theory. The high toxicity of the specimens found may limit predation of the introduced *C. simula* by native species which may facilitate establishment and spread. Further understanding of the impact this species may cause is needed to inform policy on required actions to manage the species.

### 3.2. Microbiome

The original findings of TTX-producing bacteria in marine organisms associated with TTX-presence [81,82], have not yet led to the discovery of the full biosynthetic pathway for TTX [42]. Since this time many bacterial groups have been reported as potential producers of TTX, including *Bacillus*, *Pseudomonas*, *Aeromonas*, *Vibrio*, *Actinomyces*, *Serratia*, *Shewanella*, *Microbacterium*, *Roaultella terrigenous* [83,84] with over 150 TTX-producing strains now described in the literature [44]. A large number and variety of organisms are also reported to be associated with TTX-producing bacteria, including fish, bivalve molluscs, gastropods, flatworms and echinoderms [35,44,85]. A high relative abundance was detected in the *C. simula* sample for *Alteromonas* (5.8%) *Vibrio* (2.5%), *Pseudomonas* (2.2%), which have all been described as associated with TTX production. Other genera candidates for TTX producers, such as *Shewanella* and *Serratia* [62], were also detected in lower abundance in the sample (0.6% and 0.2% respectively). The presence of a new TTX-producing *Bacillus* strain from the *C. simula* organism isolated from the waters around Japan was reported during 2012 [55]. There has also been tentative evidence for the production of TTX-like substances by *Vibrio* species isolated from other British nemerteans, such as *C. rufifrons* and *L. longissimus* [68].

By far the most commonly reported bacterial genus associated with TTX production is that of *Vibrio*, followed by *Bacillus*, *Psuedomonas*, *Actinomyces* and *Micrococcus*. Consequently, the bacteria identified from *C. simula* are all associated historically with some evidence of TTX-production. With *Vibrio* being the first bacterial genus associated with TTX-production [82], the increased focus of researchers on *Vibrio* strains may in part explain the greater number of reports of *Vibrio* strains associated with toxin presence. In this study, the *Pseudomonas lutiola* isolated from *C. simula* was found to produce toxins when cultured, providing further evidence for the association of this bacterial species with TTX-production. Interestingly, the TTX-negative nemertean was found to contain *Vibrio alginolyticus* which in turn was also found to produce toxin, potentially suggesting that TTX-producing bacteria are living symbiotically with certain species of nemertean, even if the worms themselves are not found to contain toxins. We therefore have shown evidence for toxin production from bacterial strains which are present in toxin-free higher marine organisms, suggesting the links between bacteria, higher level marine organisms and toxin presence are highly complex. The presence has been reported of a non-toxic low molecular weight compound produced by *Vibrio alginolyticus* isolated from *L. lonissimus* which could be incorrectly interpreted as the neurotoxin [61]. Given the high specificity of the instrumental detection methods used for TTX, however, we have strong evidence for TTX-production or association with both *Pseudomonas lutiola* and *Vibrio alginolyticus.*

### 3.3. Toxicity and Toxin Profile

The *C. simula* sample reported in this study, was found to contain a total summed concentration for TTX analogues of 54.3 µg per gram of nemertean tissue, equating to 54,300 µg/kg. As such, the toxin levels within this sample is very high, in comparison to the lower levels quantified to date in English bivalve molluscs, where the maximum concentration has been reported as 253 µg/kg [37]. In countries where toxic ribbon worms have been detected, potencies were found to vary enormously between specimens, with the total toxicity of ribbon worms from Japan reaching 5050 µg/g in *C. lineus* [53] assuming a conversion factor of 0.2 TTX eq per mouse unit (MU) [86] or 5555 µg/g when using the 0.22 TTX eq per MU conversion stipulated in the Japanese Official MBA method for TTXs [87]. Other authors have previously reported high neurotoxicity following MBA in the ribbon worms *Lineus fuscovirdis* and *Tubulanus punctatus*, with 100 and 109 µg TTX eq/g respectively [15]. The *Cephalothrix* species toxicity described originally by [54] was found to reach 2948 µg TTX eq/g, with the reports of *C. simula* later in Japan describing maximum TTX toxicity of nearly 5120 µg TTX eq/g tissue [14]. Consequently, the TTX concentrations in our sample of *C. simula* appear similar to those in *Lineus fuscovirdis* and *Tubulanus punctatus* but nearly an order of magnitude lower than the levels quantified by bioassay in *Cephalothrix* species originating from Japan.

The reasons for the presence of TTX in these nemertean species are not yet fully understood, although links between marine and intestinal bacteria in a variety of marine organisms are well reported [42,82,83,84,88,89,90]. This paper reports the first physical finding of *C. simula* in the UK, so further work is required to determine the likelihood of this species occurrence and the variability in toxicity between nemerteans found in the UK. In nemertean studies describing TTX toxicity in multiple specimens from within one geographical area, a seasonal variation was reported throughout the year, with a size dependency proposed [53] but not confirmed by other researchers [14]. Variability in toxin levels has also been determined through the bodies of ribbon worms with [53] reporting highest toxicity per gram of tissue in the proboscis, whereas total lethal potency was highest in the body of *C. lineus* due to the highest tissue weight. The presence of the highest concentrations of toxin in the proboscis has led some authors to propose the use of this organ as a prey capture mechanism [53,91]. Later work confirmed the presence of TTX in both the body wall and the proboscis [60]. The presence, however, of toxins in bacteria isolated from within nemertean tissue, suggests that multiple factors may be relevant. Potentially therefore, some species of nemerteans may be associated with endogenous TTX-production, with others containing TTX-producing symbiotic bacteria. Further work will be required to assess the relationships between the nemerteans, bacteria and toxins in order to fully understand these relationships once more toxic nemerteans are obtained.

LC-MS/MS data confirmed the parent TTX as being the dominant analogue, as found with the toxin profile in all English bivalve samples to date [37]. The bivalve samples, however, were not generally found to contain the wide range of other analogues detected here in the nemertean, with the *C. simula* showing at least nine other analogues at quantifiable concentrations. Similarly, ribbon worms from Japan have also been found to contain multiple TTX analogues [54]. Toxicity equivalence factors (TEFs) describing relative potency have not, however, been formally assigned for TTX analogues due to a high level of uncertainty in methods used to derive these values. Nevertheless, for some analogues, TEFs have been suggested, including in order of potency, 11-oxo TTX (0.75), 11-nor TTX-6-ol (0.18), 4-epi TTX (0.16), 11-deoxy TTX (0.14), 4,9-anhydro TTX (0.02), 6,11-dideoxy TTX (0.02) and 5,6,11-deoxy TTX (0.01) [92]. With application of these relative potencies and assuming a TEF of 1 for analogues present in the nemertean with unknown values as a worse-case scenario, the *C. simula* sample from Cornwall contains a total TTX equivalence of 37,027 µg TTX eq/kg. Assuming the toxicity of TTX is 91% of saxitoxin (STX) [86], then this total nemertean toxicity equates to 33,660 µg STX eq/kg, more than 42 times higher than the EU regulatory limit for PSP toxins in bivalve mollusc shellfish [93].

### 3.4. Human Safety

With reported levels of TTXs in one single *C. simula* organisms exceeding the minimum lethal dose of TTX [14], there are risks from this species if the worms become introduced to shellfishery products, or the worms become ingested through other means [67]. The risk from TTX-positive nemerteans entering the food chain through trophic feeding therefore needs careful assessment. Previous reports suggest toxin transfer resulting in the toxicity of trumpet shells following feeding on toxic starfish [88,90]. In Japan, *C. linearis* (subsequently found to be easily confused with *C. simula*, [14]) was found to release toxins when lightly stimulated with the hands. Out of 52 stimulation experiments conducted, secreted toxin concentrations ranged from approximately 1 to 80 TTX equivalence per individual specimen, meaning that significant concentrations of toxins could be imparted to humans if handled, although the risks would only be heightened if the secreted toxins were subsequently ingested [53]. In Japan, highly toxic ribbon worms were described as adhering to oysters grown on floating rafts in aquaculture systems [54], thereby providing a risk of contamination in oysters from these species, although the authors described these as being fixed only to the surface of the shellfish shells. Subsequently, shucked oyster meat was reported as being safe to eat, as the nemerteans are removed from the outer shell during the washing step [14].

In the one *C. simula* sample from this study, the total TTX toxicity was significantly lower than that determined from worms of the same species in Japan, thus reducing the overall level of risk in England significantly, based purely on the results from this study. Following the collection of 764 nemerteans in Japanese waters for toxicity determination, the average weight was 0.36 ± 0.30 g per specimen, 80% of which were found to be highly toxic, above 164 µg TTX eq/g tissue [14]. Consequently, a single worm with a weight even as high as 1 g being ingested, based on the toxicity determined in the *C. simula* worm from this study, would result in the consumer being exposed to approximately 50 µg TTXs. Acute toxicity from TTX is not well defined but human case reports indicate poisoning can result from doses of 4–42 µg TTX per kg of body weight, relating to a minimum of 200 µg TTX in a 50 kg human [92]. Whilst the 50 µg TTX present in a 1 g nemertean, based on the findings from the one worm described in this study is below this range, the absence of data regarding prevalence and toxicity in a larger number of species, given the high variability in toxicity recorded in previous studies form Japan, makes further study in this area important.

Overall, whilst the hazard from TTX presence in this non-native nemertean species is clear, the level of risk is currently impossible to define and a review is advised to enable more data to be generated on worm presence, distribution and toxicity in the natural marine environment within English coastal waters, as well as potential uptake of the toxins into higher level organisms. Likewise, this applies to determining any further environmental risk that the species may pose. More sampling will be required in particular to determine the likely numbers and geographical spread of *C. simula* in the marine environment of the English coast and to measure the levels of toxicity across populations. The sampling studies should also help determine whether the nemerteans are in danger of contaminating live bivalve mollusc shellfish or other fishery products either directly or through trophic transfer, as presently there is no evidence that this risk is present. This information will aid policy in prioritising decision making on how or if the species should be managed in the future.

## 4. Materials and Methods

### 4.1. Reagents and Chemicals

For toxin analysis of all sample types, instrument solvents, test reagents and chemicals were either HPLC or LC-MS grade as appropriate to the assay. Certified reference material (CRM) for tetrodotoxin used for preparing instrumental calibrants were obtained from Cifga (Lugo, Spain). A TTXs-positive freeze-dried tissue of the sea slug *Pleurobranchaea maculata* was purchased from Cawthron Natural Compounds (CNC; Nelson, New Zealand) and used for preparation of an internal quality control material enabling identification of TTX analogue retention times. A TTX stock standard solution was prepared from the Cifga CRM and used to prepare working standards over six concentration levels for quantitation by external calibration. Calibrants were prepared by diluting a stock mix in 80% acetonitrile (MeCN) with 0.25% acetic acid. Quantitation of all TTX analogues was performed against the TTX calibration, given the low relative concentration of other analogues present in the certified standard. Analysis of concentrated CRM solution diluted by a factor of 10, enabled the qualitative detection of the minor analogues which enabled the confirmation of additional analogues in QC samples, thereby facilitating confirmation in unknown samples.

For microbiological analyses selective agars Thiosulphate-citrate-bile salts-sucrose agar (TCBS Oxoid) and BioMérieux chromID™ *Vibrio* agar (VID) were used for bacterial isolations direct from neat worm matrix and from the following enrichment broths: Nutrient Broth (NB Oxoid) and Alkaline salt peptone water (ASPW Oxoid). Presumptive colonies were selected based on colony morphology and colour, then sub-cultured on to Marine agar (DIFCO). Pure cultures were subsequently identified to species level using API20E (Biomerieux, Marcy l’Etoile, France).

### 4.2. Species Confirmation of Nemertean

A sample of the nemertean worm 1b (Table 1) preserved in ethanol was split into two and run through parallel DNA extractions. Samples were homogenised in Lysing matrix A tubes in a Fast Prep system (MP biomedicals, Santa Ana, CA, USA, https://www.mpbio.com). DNA extraction was done with Sigma GenElute Mammalian Genomic DNA Miniprep Kit following manufacturer’s protocol (Sigma, Poole, UK, https://www.sigmaaldrich.com/). Amplification of barcoding gene Cox 1 was done by PCR, with 2 µL of DNA from each sample used as template in standard 50 µL reactions with previously published primers at 0.4 µM (mlCOIint 5′-GGWACWGGWTGAACWGTWTAYCCYCC-3′ and HCO2198 5′-TAAACTTCAGGGTGACCAAAAAATCA-3′; [94,95] respectively). PCR was run on a BioRad Tetrad thermocycler (BioRad, Liverpool, UK, www.bio-rad.com) with a single melting step of 94 °C for 2 min; 35 amplification cycles of 94 °C for 30 s, 45 °C for 30 s and 72 °C for 1 min; and a final elongation step at 72 °C for 5 min [94]. Amplified samples were cleaned by Wizard SV Gel and PCR Clean-Up System according to manufacturer’s protocol (Promega, Madison, WI, USA, https://www.promega.co.uk) before being sent for single read sanger sequencing at Eurofins genomics (Eurofins genomics, Ebersberg, Germany, https://www.eurofinsgenomics.eu). DNA sequences were trimmed manually to remove primer sequence contamination and any poor-quality data before being run through blastn against the nr database.

### 4.3. Microbiological Culturing and Testing

For microbiological analyses 10 µL of homogenised worm were sub-cultured onto selective agar Thiosulphate-citrate-bile salts-sucrose agar (TCBS) and BioMérieux chromID™ Vibrio agar (VID). The remaining subsample of homogenised worm were enriched using Nutrient Broth (NB) and Alkaline salt peptone water (ASPW) at multiple temperatures 22 °C, 37 °C and 41.5 °C and incubated for 24/48 h. The enrichment broths were then also sub-cultured onto TCBS and VID. Presumptive colonies were selected based on colony morphology and colour, then sub-cultured on to Marine agar (DIFCO) and incubated at 30 °C. Pure cultures were subsequently identified to species level using API20E (Biomerieux, Marcy l’Etoile, France).

Initial streak plates were dominated by presumptive *Vibrio alginolyticus* with few opportunities for clean isolations. Stored enrichment broths (Room temperature in the dark) were sub-cultured on multiple plates resulting in improved isolations.

Colonies of presumptive *V. alginolyticus* strains were subsequently analysed by PCR, using primers recognising two separate *V. alginolyticus* species-specific targets, collegenase and the DNA replication gene gyrB, essentially as previously described [96,97].

### 4.4. Microbiome Sequencing

Preparation of the metagenomic library was based on 16S rRNA gene amplicons. Amplification of the 16S rRNA gene was carried out with the16S Barcoding kit SQK-RAB204 of Oxford Nanopore Technologies using the Master mix LongAmp taq 2X (NEB) following the instructions provided by Oxford Nanopore Technologies. PCR products were purified with the AMPure XP beads kit (Beckman Coulter, Brea, CA, USA) and quantified using the Qubit dsDNA BR Assay kit (Invitrogen, Merelbeke, Belgium) with a Qubit 2.0 Fluorimeter. To optimize the output of the MinION platform, a multiplexing strategy was applied using the 16S Barcoding kit SQK-RAB204 (ONT). The library was prepared according to the manufacturer’s instructions and sequencing was carried out on a MinION sequencer (ONT) using a R9.4 flowcell. For bioinformatic analysis, Albacore 2.3.1 was used for the basecalling and conversion of the raw data to FASTQ format. Taxonomic assignment was carried out using the bioinformatic tool Centrifuge 10.3-beta [98]. Plots and analysis of taxonomic abundance were made with Pavian 0.3 (https://github.com/fbreitwieser/pavian) and PHINCH (Bik, 2014) (https://github.com/PitchInteractiveInc/Phinch).

### 4.5. Toxin Testing

#### 4.5.1. Nemertean Tissues

Analysis of tetrodotoxin (TTX) and its associated analogues (TTXs) was conducted using a validated ultra-high-performance liquid chromatography with tandem mass spectrometry method (UHPLC-MS/MS, here abbreviated further to LC-MS/MS) [99]. For Worm 1a, 275 mg of homogenised nemertean tissue was weighed into a 2 mL polypropylene Eppendorf tube. 550 µL 1% acetic acid was pipetted into the tube and the contents vortex mixed for 90 s, followed by 2 min ultrasonication and an additional 90 s vortex mix. A procedural blank (PB) consisting of 275 µL deionised water and 550 µL 1% acetic acid was treated using exactly the same process. For Worm 3, 33 mg of homogenised tissue were extracted using 264 µL 1% acetic acid. Worm 2, which presented as partially-decomposed tissue on a sponge in seawater was treated differently. Half of the sponge sample was added to a 15 mL centrifuge tube and extracted using 5 mL 1% acetic acid. Samples were then extracted using a scaled down version of the validated TTX method [99] depending on the size of the samples. Eppendorf tubes (Worm 1 and Worm 3) and centrifuge tube (Worm 2) were sealed and placed into boiling water for 3 min, before cooling under running water for a further 3 min. Tube contents were micro-centrifuged for 10 min at 10,000 rpm. The supernatant was subsequently subjected to graphitised carbon solid phase extraction (SPE) using Supelclean Envi-carb SPE cartridges, prior to dilution in acetonitrile prior to LC-MS/MS analysis as described by [99].

HILIC-MS/MS analysis was conducted using an Agilent (Manchester, UK) 1290 UHPLC coupled to an Agilent 6495B tandem quadrupole mass spectrometer (MS/MS) with methods undertaken as described by [99]. Each instrumental sequence started with a start-up inlet and finished with shutdown inlet methods as detailed by [99,100]. MRM transitions were taken from [99]. Analysis incorporated the direct quantitation of TTX against the external TTX calibration. Primary (quantitative) and secondary (qualitative) MRMs for each analogue were as follows: TTX and 4-epi-TTX (320.1 > 302.1, 162.1), 5,6,11-trideoxy TTX (272.1 > 254.1, 162.1); 11-nor TTX-6-ol (290.1 > 272.1, 162.1); 4,9-anhydro TTX (302.1 > 256.1, 162.1); 5-deoxy TTX (304.1 > 286.1, 176.1), 11-deoxy TTX (304.1 > 286.1, 176.1), 4,9-anhydro-5,6,11-trideoxy TTX (254.1 > 236.1, 162.1), 11-oxo TTX (336.1 > 318.1, 162.1). Transitions for arginine and hydroxy-arginine were also assessed following [99], in order to evidence effective chromatographic separation of TTX from these matrix co-extractives, which are known to affect TTX quantitative recovery and therefore method accuracy. MRMs for all additional analogues were acquired, with semi-quantified concentrations determined using the TTX calibration. The presence of the additional TTXs analogues was carried out by comparing both quantitative and qualitative MRM peaks and their associated ion ratios against those established in the concentrated calibration standard and the quality control extract. The Limit of Detection (LOD) and Quantitation (LOQ) for the method [99] were found to improve through use of the Agilent MS/MS, resulting in LOD and LOQ of 0.2 and 0.7 µg/kg respectively for TTX based on a standard 1:1 single dispersive extraction procedure. No recovery correction or adjustment for toxic equivalence was made.

#### 4.5.2. Bacterial Cultures

50 mL cultures of bacteria were centrifuged for 10 min at 4500 rpm, the supernatant removed and the bacterial pellet extracted with the addition of 1 mL 1% acetic acid and vortex mixing (1 min). Pellets were left in solution for 1 h prior to a second vortex mix. Solutions were then placed into boiling water for 3 min, before being cooled in running cold water for 3 min, vortex mixing (1 min) and centrifugation (10 min, 4500 rpm). The supernatant was transferred to an Eppendorf tube and re-centrifuged (10 min, 10,000 rpm) before removing the supernatant and subjecting to carbon SPE clean-up and dilution with acetonitrile. Cleaned-up and diluted extracts were analysed by HILIC-MS/MS as described above.

## Figures and Tables

**Table 1:** Summary of samples received for study.

Sample	Matrix ^a^	Date Collected	Location
Worm 1a	*C. simula* (in seawater)	18 May 2018	Godrevy Point, Cornwall
Worm 1b	*C. simula* (in ethanol)	18 May 2018	Godrevy Point, Cornwall
Worm 2	*C. rubifrons* (in seawater with sponge)	29 May 2018	Skilly, Newlyn, Cornwall
Worm 3	*T. annulatus* (in seawater)	29 May 2018	Skilly, Newlyn, Cornwall

^a^ Tentatively speciated through morphological identification.

**Table 2:** Relative abundance of bacterial genera identified in *C. simula*. TID: Taxon ID.

Name	TID	Relative Abundance	Sample1	Sample2
*Alteromonas*	226	5.8055	7.102	4.509
*Ehrlichia*	943	4.1325	5.933	2.332
*Edwardsiella*	635	2.94	3.322	2.558
*Vibrio*	662	2.5565	2.579	2.534
*Wolbachia*	953	2.524	3.383	1.665
*Colwellia*	28,228	2.3225	1.933	2.712
*Pseudomonas*	286	2.2095	2.206	2.213
*Escherichia*	561	2.0995	2.034	2.165
*Auricoccucs*	2,005,363	1.983	2.717	1.249
*Sulfitobacter*	60,136	1.8645	1.481	2.248

**Table 3:** Summary of TTXs concentrations (ng/mL of extract and ng/g tissue) in *C. simula* nemertean sample 1a, highlighting percentage proportion of overall toxin concentrations per TTX analogue.

	TTX	6,11-dideoxy TTX	5,6,11-trideoxy TTX	11-oxo TTX	5-deoxy TTX/11-deoxy TTX	4,9-anhydro TTX	11-nor TTX-6-ol	4,9-anhydro-5,6,11-trideoxy TTX	4,9-anhydro-11-oxo TTX
Retention time (min)	6.17	3.65	2.53	7.13	4.33/5.32	4.39	5.04	2.31	4.68
ng/mL	580	186	86	41	8.4	3.2	0.57	0.47	0.21
ng/g	34,791	11,170	5146	2454	504	193	35	28	12
Proportion	64%	21%	9%	5%	0.9%	0.4%	0.1%	0.1%	0.02%

## Supplementary Materials

The following are available online at http://www.mdpi.com/1660-3397/16/11/452/s1, Figure S1: Sequences from two worm fragments.
