# Supplementary material for: New Invasive Nemertean Species (Cephalothrix Simula) in England with High Levels of Tetrodotoxin and a Microbiome Linked to Toxin Metabolism
Source: Mar Drugs. 2018 Nov 16;16(11):452. doi: 10.3390/md16110452 (PMC6266807; doi:10.3390/md16110452)
Supplement: Supplementary file 1 [file marinedrugs-16-00452-s001.pdf]

Supplementary Material

Figure S1. Cox1 DNA barcode sequences of *C. simula* (worm 1b).

Worm fragment 1:
GATCTCCCCCTCCCGCAGGATCAAAAAAAGAAGTATTAAAATTTCGATCAGTTAATAATATA
GTAATTGCACCAGCTAATACTGGTAAAGATAATAATAATAAAATAGCAGTAATTTTTACAGA
TCAAACAAACAAAGGAAGACGTTCAAAATGTATACCTCGTCATCGCATATTAATAATAGTTG
TAATAAAATTAATAGCTCCTAAAATTGAAGAAACTCCAGCTAAATGCAAAGAAAAAAATAGC
TAAATCTACAGAACCTCCAGCATGAGCTAAATTTCCTGATAAA

Worm fragment 2:
GATCTCCCCCTCCCGCAGGATCAAAAAAAGAAGTATTAAAATTTCGATCAGTTAATAATATA
GTAATTGCACCAGCTAATACTGGTAAAGATAATAATAATAAAATAGCAGTAATTTTTACAGA
TCAAACAAACAAAGGAAGACGTTCAAAATGTATACCTCGTCATCGCATATTAATAATAGTTG
TAATAAAATTAATAGCTCCTAAAATTGAAGAAACTCCAGCTAAATGCAAAGAAAAAAATAGC
TAAATCTACAGAACCTCCAGCATGAGCTAAATTTCCTGATAAA
